# We need to talk about the virome

**DOI:** 10.20517/mrr.2026.13

**Published:** 2026-06-26

**Authors:** Colin Hill

**Affiliations:** APC Microbiome Ireland and School of Microbiology, University College Cork, Cork T12YT20, Ireland.

Studying the composition and functionality of the human microbiome is a very complex task. Most researchers simplify this by focusing on the faecal bacteriome, either by 16S rRNA analysis, or by extracting bacterial reads following shotgun sequencing. Here I suggest that the gut virome, dominated by bacteriophages (phages), should not be treated as a footnote to the bacteriome but should be integrated into how we think about microbiome structure, function and its impact on host health. Drawing largely from our own work over the last decade, I will argue that phages are important contributors within gut microbial ecosystems rather than incidental passengers.

**An overview of the virome**. There are an estimated 10^14^ phages in the gut, with genome sizes ranging from a few kilobases to over 500 kb and encompassing all major nucleic acid types (single or double stranded RNA, single or double stranded DNA) and many different morphologies and lifecycles^[1,2]^. Lytic (infect, replicate, lyse) or lysogenic (infect, integrate, co-exist) lifecycles predominate, but other options also exist. Only a tiny fraction of these phages has ever been isolated or studied in a laboratory and most phage genomes are now identified *in silico* following metagenomic sequencing of total microbiomes or of crudely extracted viral particles. Our understanding of phage biology is almost completely shaped by those phages that form visible plaques on lawns of cultivatable bacteria, but we know that there are many phages that do not form plaques, and there are many bacteria that do not form lawns. This means that our understanding of the virome is increasingly shaped by bioinformatics with very limited wet-lab input, with all the attendant challenges that presents in terms of determining lifecycles, hosts and functional inference. Phage taxonomy is also highly complex because phages do not have the equivalent of a universal marker gene like that encoding 16S rRNA in bacteria. As an example of this taxonomic challenge, crAssvirales is the most abundant known order in the human gut but phages within the order can share extremely low DNA identity and some families even use alternative genetic codes^[3,4]^. Such variation within a single order challenges traditional sequence-centric notions of what constitutes a phage “species” or even a coherent taxonomic unit. It is worth noting that recent bioinformatic advances are helping to resolve taxonomic structure, with programmes like vCONTACT2^[5]^, vCONTACT3^[6]^ and SinProVirP^[7]^ pointing the field towards a future where robust taxonomic assignments may prove feasible. In addition, protein structure approaches will significantly improve functional inference for phage genes, particularly for distantly related homologs^[8,9]^.

Most studies to date have focused on the faecal virome, but humans and animals have multiple viromes across mucosal and extra-intestinal sites (skin, tongue, stomach, small intestine segments, caecum, colon regions, liver, lung, spleen)^[10]^. We also must consider that spatial structure and compartmentalisation matter for both phage ecology and host interactions. Longitudinal data confirms that each person carries a distinctive and relatively stable virome and supports the idea that individual phages or virome composition could act as biomarkers of microbiome structure and function, or even as sentinels of disease states^[11]^. The temporal stability of the human virome raises some intriguing questions of how it is maintained in the face of constant environmental and dietary perturbations, and to what extent it is a property of the phage themselves versus their bacterial hosts and their host environment?

**Fighting or dancing?** Perhaps some of the answers of how phage and their target bacteria co-exist in the microbiome can be gleaned from investigating specific phage-bacteria interactions in species where phage biology has not previously been studied to any great extent. One example that illustrates the benefits of studying phage in culture rather than *in silico* are the aforementioned crAssvirales. The cultivation of the first member of the order, φcrAss001 infecting *Bacteroides intestinalis *in 2019, has allowed us to study this phage in detail^[12]^. High-resolution cryo-EM structural work underscores the sophistication of the phage structure and of phage-host interactions at the cell surface and envelope^[13]^. This connects directly to issues of host range, sensitivity and insensitivity, and informs us as to how phage can potentially be used as a tool in microbiome science to understand and even manipulate microbiome-host dynamics.

At the single-cell level, most phage-bacteria interactions can be described as a fight to the death. Either the phage ‘wins’ through adsorption, infection and lysis of its bacterial target, or the bacterium ‘wins’ by using one of a plethora of resistance mechanisms to ensure that no viable phage progeny result from the initial contact. Lysogeny could be described as a draw, but one where the possibility of phage-induced lysis remains a threat. Yet at the population level in complex, structured communities, the evidence points towards coexistence of both lytic and lysogenic phages and their host bacteria. Mechanisms include cycles of resistance and sensitivity, lysogeny, chronic infections, and dynamic equilibria that collectively sustain both phage and host populations. This can be better described as an ongoing “dance” where highly lytic phages can coexist with their permissive hosts over long periods *in vitro* and *in vivo* without causing the collapse of the bacterial population. This dance can represent a win-win scenario for both phage and bacterial host, with both parties benefitting from the interaction. It is worth remembering that no phage currently in existence has ever completely eliminated its bacterial host(s), and very few (if any) bacterial species do not have associated phages.

φcrAss001 and *B. intestinalis* provides a concrete example of this coexistence. In mono-associated mice, the phage maintains stable titres over more than 100 days without measurably reducing bacterial counts, with both phages and bacteria persisting at high levels^[14]^. This challenges simplistic assumptions that more phages will lead to fewer bacteria or that the target bacterium will become completely resistant leading to the elimination of the phage but supports models in which phage primarily shape bacterial physiology rather than total biomass. Cycling between resistance and sensitivity, with resistant clones (altered surface structures, non-adsorbing) expanding in the presence of phage and then contracting over longer timescales, illustrates how dynamic this balance can be. Even in the absence of phage a substantial fraction of bacteria becomes resistant over time, underlying the fact that phase variation and surface diversification are intrinsic features of the bacterium, not merely phage-induced artefacts.

Transcriptomics and long read sequencing have revealed that invertible promoter-mediated regulation of operons encoding capsular polysaccharides (CPSs) causes “costume changes” that results in every population of target bacteria including both permissive and non-permissive variants^[14]^. The presence of a specific phage selects which surface polysaccharides variants escape predation, effectively reshaping the antigenic CPS “costume” of the bacterial population and allowing phage and bacterial host to reach a form of homeostasis^[15]^. This is an elegant example of how phage pressure can reward bacterial phase variation, with all the potential implications for niche occupancy, immune evasion, and susceptibility to other phages or antimicrobials. It also reminds us that “resistance” is not a static, all-or-nothing trait but is often mediated by reversible switches that carry ecological trade-offs. An elegant study performed in *Bacteroides thetaiotaomicron* confirmed that in murine gut colonisation models phase-variable strains capable of switching between eight CPS gene clusters out-perform isogenic acapsular strains or variants ‘locked’ into expressing any one of the eight CPSs^[16]^.

**Phage-mediated horizontal gene transfer**. It has long been established that phages can package bacterial DNA and deliver it to permissive recipients. There are several types of this phage-mediated transduction; including generalised, specialised and lateral^[17]^. There are also other types of capsid-mediated mobilisation of bacterial genomic DNA such as that mediated by gene transfer agents (GTAs)^[18]^. These are viral-like particles composed of portal and major capsid proteins that can package and disseminate bacterial DNA at scale in the gut. This means that the virome is not just a top-down predator shaping bacterial composition but also acts as an instrument for gene flow within the microbiome. In the context of antimicrobial resistance, metabolic versatility, and virulence factor dissemination, this is potentially transformative and has significant evolutionary implications. Phages also offer potential tools for precise ‘editing’ of bacterial genomes within microbiomes, for example by introducing new auxiliary metabolic genes, or by introducing CRISPR-based editing systems to remove genes^[19]^.

**Conclusions**. The virome can be a useful resource in several different ways to those wishing to understand or manipulate the microbiome. In addition to providing potential biomarkers of microbiome structure and function, phage can impact the functionality of individual bacterial strains. The virome can also mediate gene transfer (transduction/GTAs), and can potentially influence microbiome assembly, structure and function, all ultimately feeding into host health [Figure 1]. The virome can be considered as both a biomarker of microbiome state and as an active driver of change. We need to switch from viewing phage genomes as additional points of interest in metagenomic datasets to treating them as essential components of any serious microbiome analytical pipeline.

**Figure 1:**
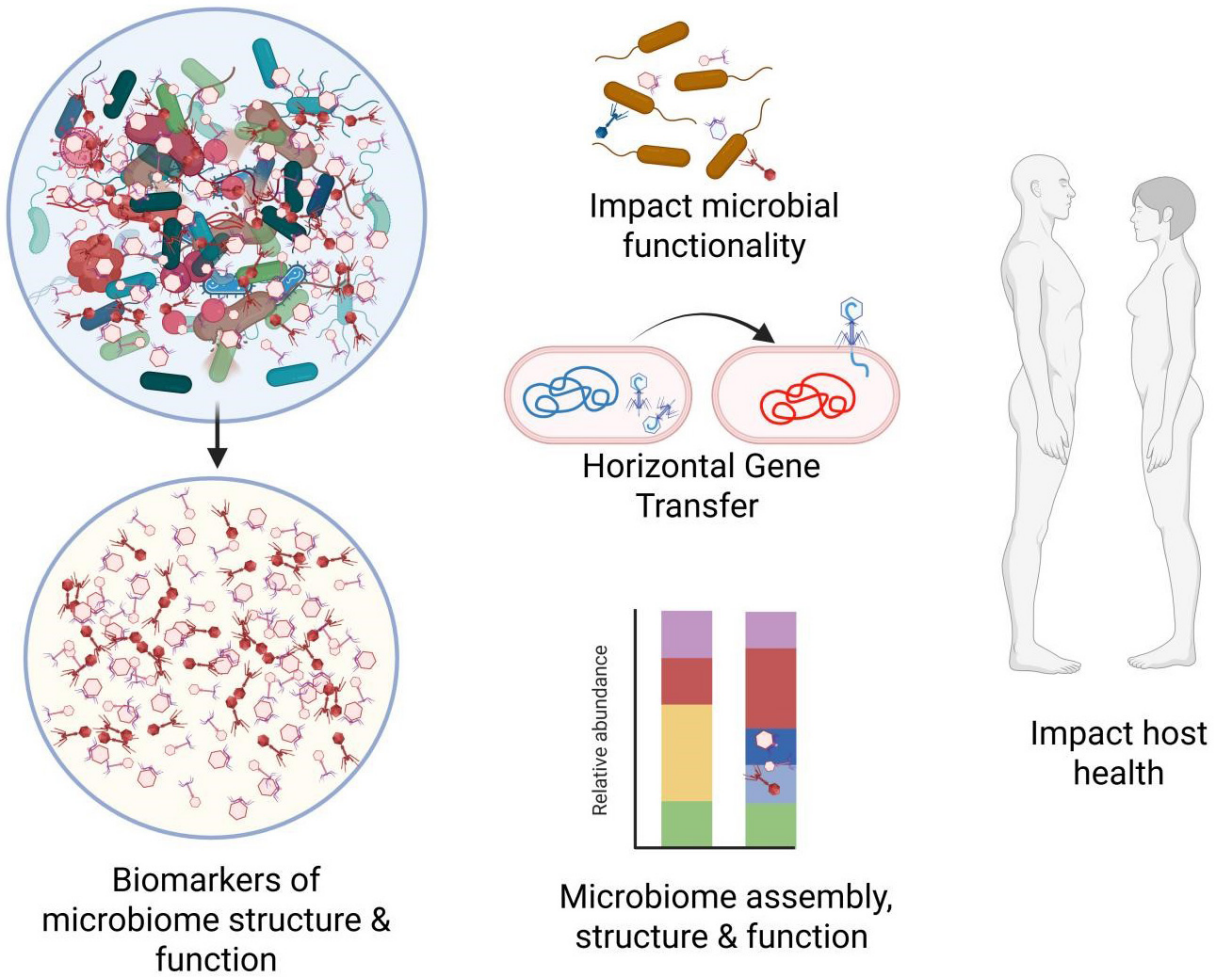
Role of the virome in human health. The virome can act as a biomarker of microbiome composition and functionality, individual phages can influence bacterial functionality, be an agent of horizontal gene transfer, or impact on microbiome assembly structure and function. All of these can ultimately impact on human health. Created in BioRender. Hill, C. (2026) https://BioRender.com/pttw89l

## DECLARATIONS

### Copyright

© The Author(s) 2026.
